# Regional excess mortality in France during COVID-19 pandemic: the first three epidemic periods (March 2020–June 2021)

**DOI:** 10.1093/eurpub/ckae032

**Published:** 2024-02-22

**Authors:** Marlène Faisant, Nicolas Vincent, Bruno Hubert, Alain Le Tertre

**Affiliations:** Santé publique France, SpFrance, Saint-Maurice, France; Santé publique France, SpFrance, Saint-Maurice, France; Santé publique France, SpFrance, Saint-Maurice, France; Santé publique France, SpFrance, Saint-Maurice, France

## Abstract

**Background:**

This study aimed to describe the mortality excess during the three first epidemic periods of COVID-19 in all regions of France.

**Methods:**

Two complementary approaches were implemented. First, we described the number of death of patients infected with or diagnosed with COVID-19 in health care (HC) and medico-social (MS) institutions. Then, we estimated general all-cause mortality excess (all ages) by comparing the mortality observed with the expected mortality. We used a daily number of death model according to a negative binomial distribution, as a function of the long-term trend in mortality (penalized spline function of time) and its seasonal variation (cyclic spline function). The model provided expected mortality during epidemic periods with a 95% credibility interval. Each region defined three epidemic periods, including the overseas territories.

**Results:**

The two approaches were consistent in the most affected regions but there are major regional disparities that vary according to the epidemic period. There is an east–west gradient in the relative excess of deaths from all-causes during each epidemic period. The deaths observed in HC and MS institutions alone do not explain the excess (or deficit) of mortality in each region and epidemic period.

**Conclusion:**

An analysis by age group according to the two approaches and a comparison of death specific causes could provide a better understanding of these differences. Electronic death registration system (mortality by medical causes) would allow a rapid mortality related estimation to an emerging pathology like Coronavirus Disease-2019 (COVID-19) but is still insufficient for real-time medical causes of death monitoring.

## Introduction

The SARS-CoV-2-related pandemic began in Wuhan, China in late 2019. The virus spread rapidly around the world in early 2020. In France, the first cases of severe acute respiratory syndrome coronavirus 2 (SARS-CoV-2) were detected in late January 2020.^1^

In order to monitoring this pandemic in France, Santé publique France, the French national public health agency, has several surveillance systems. Deaths related to COVID-19 are estimated from health care (HC) and medical-social (MS) institution data. At the same time, all-cause mortality is monitored in near-real time using data from computerized civil-status offices transmitted to the National Institute of Statistics and Economic Studies (INSEE).^2^ It does not allow the description of medical causes of death. These are coded, monitored and analyzed by the French epidemiological centre for medical causes of death (CépiDC-Inserm), which requires a lengthy transcription process.^3^ They were not available for the study period at the time of analysis.

The aim of this article was to assess direct or indirect mortality associated with the COVID-19 pandemic in the French regions, during the first three waves occurring in France between March 2020 and May 2021, by synthesizing the results presented regionally elsewhere.^4^

## Data

Three complementary data sources were analyzed.

### SI-VIC

The information system for monitoring victims of attacks and exceptional health situations (SI-VIC) aims to identify and follow the hospital course of victims of an exceptional event.^5^ As part of the COVID-19 pandemic, HC providers must enter biologically positive COVID-19 patients into this database on a daily basis, regardless of the purpose of their hospitalization: every event from their hospitalization until their return home or death was updated. Data on hospital deaths were collected from the start of the epidemic (13 March 2020) with a ramp-up to 23 March 2020.^6^ Deaths, regardless of cause, are assigned to the first hospitalized region.

### Social and medico-social institutions

From March 2020, the MS institutions have been reporting the deaths of their residents linked to COVID-19 *via* a specific application (COVID-19 EHPAD/ESMS) developed by Santé publique France and accessible *via* the reporting portal.^7^ The data collected concern all COVID-19 resident deaths occurring in MS institutions between 2 March 2020 and 6 June 2021.

### INSEE—deaths from all-causes

All-cause deaths in a panel of about 3000 municipalities are transmitted to INSEE in near real time by the civil-status offices.^3^ On an average, these municipalities account for 77% of deaths in France. This rate varies at the regional level. The data are consolidated at 30 days and are then exhaustive for these municipalities. The data collected, daily numbers of deaths of all ages, cover the period from 1 January 2015 to 15 July 2021. These daily data are then adjusted at the regional level to the entire population by applying an exhaustiveness coefficient for each region.

## Methods

To assess mortality during the COVID-19 epidemic, two complementary approaches were implemented.

### First approach

The first was to summarize COVID-19-identified deaths that occurred in HC and MS institutions. The aim was to give the number of deaths of patients infected with or diagnosed with COVID-19. However, only deaths that occurred in HC or MS institutions were included, leaving out deaths at home.

### Second approach

The second was to estimate general all-cause mortality (all ages) by comparing the mortality observed during the different epidemic periods with the expected mortality that should have occurred during these periods in the absence of a COVID-19 epidemic. The aim was to determine whether the number of deaths observed during the epidemic periods was higher or lower than usual.

The method chosen was based on modelling the daily number of deaths at the regional level according to a negative binomial distribution, as a function of the long-term trend in mortality and its seasonal variation. The modelling of the long-term trend was based on a penalized spline function of time with a number of degrees of freedom defined *a priori* at 7, as the number of years of available data. The seasonal component was defined *via* a cyclic spline function with a number of degrees of freedom also defined *a priori* at 12, corresponding to the number of months of the year. Epidemic periods were specifically modelled using spline function and with sufficient flexibility to capture variations during these periods. Thus, the parameters of interest, long-term trend and seasonality, were not influenced by these specific periods. In addition, a weekday effect was taken into account. The model provided expected mortality during epidemic periods with a 95% credibility interval, incorporating the uncertainty associated with the selection of smoothing parameters for the epidemic periods. This expected mortality, as well as the lower and upper bounds of its credibility interval, was subtracted from the observed mortality during these periods to determine the number of excess or deficient deaths. The analyses were performed in R^®^ V4.0.4 (*mgcv* package).

### Epidemic periods

The regional epidemic periods used in the analysis were defined by the Santé publique France regional teams and are listed in table 1. These epidemic periods have been adapted for metropolitan France and the French overseas departments (FOD). The epidemic periods including early January 2021 were divided in two to take into account the implementation of the vaccination campaign against COVID-19.^8^^,^^9^ The third epidemic period in French Guiana was not included. Its period was, as for the next epidemics in France, concomitant with other usual epidemics.

**Table 1:** First three regional epidemic periods of COVID-19, March 2020 to May 2021 (Sources: Santé publique France)

Region	1st epidemic period	2nd epidemic period	3rd epidemic period
Metropolitan France,	2 March 2020 to 31 May 2020	21 September 2020 to 31 December 2020	1 January 2021 to 31 May 2021
Réunion Island^a^	(2020-W10 to 2020-W22)	(2020-W39 to 2020-W53)	(2021-W01 to 2021-W22)
Martinique, Guadeloupe	2 March 2020 to 31 May 2020	3 August 2020 to 15 November 2020	8 February 2021 to 31 May 2021
	(2020-W10 to 2020-W22)	(2020-W32 to 2020-W46)	(2021-W06 to 2021-W22)
French Guiana	1 June 2020 to 6 September 2020	30 November 2021 to 14 February 2021	
	(2020-W23 to 2020-W36)	(2020-W49 to 2021-W06)	
Mayotte^a^	2 March 2020 to 2 August 2020	21 September 2020 to 31 December 2020	1 January 2021 to 31 May 2021
	(2020-W10 to 2020-W31)	(2020-W39 to 2020-W53)	(2021-W01 to 2021-W22)

a The period from 21 September 2020 to 31 May 2021 has been divided in two to take into account the implementation of vaccination starting in January 2021.

## Results

All the results are summarized in table 2.

**Table 2:** Number of deaths related to COVID-19 recorded in health care (HC) and medico-social (MS) institutions and excess all-cause mortality for all ages estimated by modelling, for the first three regional epidemic periods of COVID-19, by region, March 2020 to May 2021

		HC and SMS institutions				Excess all-cause mortality								
						1st epidemic period			2nd epidemic period			3rd epidemic period		
Region	Population^a^	1st epidemic period	2nd epidemic period	3rd epidemic period	Total	Absolute excess		Relative excess (%)	Absolute excess		Relative excess (%)	Absolute excess		Relative excess (%)
Auvergne-Rhône-Alpes	8 197 325	2983	8552	5340	16 875	2684	[2254; 3104]	15	9011	[8438; 9568]	45	3818	[2852; 4754]	13
Bourgogne-Franche-Comté	2 786 296	1668	2627	2745	7040	1647	[1404; 1882]	23	2521	[2196; 2834]	30	1500	[938; 2039]	12
Brittany	3 429 882	334	602	983	1919	−281	[−543; −26]	−3	328	[−16; 660]	3	252	[−337; 819]	2
Centre-Val-de-Loire	2 572 278	890	1188	1778	3856	515	[278; 744]	8	954	[640; 1256]	13	1018	[477; 1534]	9
Corsica	351 255	72	64	90	226	67	[−16; 142]	9	59	[−54; 160]	7	−16	[−216; 160]	−1
Grand-Est	5 562 262	5352	3113	4379	12 844	4880	[4542; 5210]	38	2610	[2164; 3043]	18	2713	[1950; 3451]	12
Hauts-de-France	5 980 697	2504	3125	4825	10 454	2222	[1856; 2579]	16	2648	[2157; 3124]	17	3089	[2250; 3900]	12
Île-de-France	12 358 932	11 872	4878	8274	25 024	12 443	[12 047; 12 830]	67	3048	[2506; 3578]	14	5094	[4191; 5972]	15
Normandy	3 317 023	614	1553	2043	4210	445	[181; 701]	5	1098	[740; 1443]	11	920	[310; 1506]	6
New Aquitaine	6 110 365	523	2070	2598	5191	−229	[−617; 149]	−1	1662	[1147; 2163]	9	1104	[219; 1963]	4
Occitania	6 101 005	689	2545	2920	6154	250	[−123; 614]	2	2301	[1795; 2792]	14	1482	[623; 2314]	6
Provence-Alpes-Côte-d’Azur	3 907 426	736	1829	2101	4666	1055	[747; 1355]	9	3345	[2915; 3762]	22	3531	[2809; 4232]	16
Pays-de-la-Loire	5 160 091	1163	3136	4692	8991	385	[113; 650]	4	890	[526; 1243]	9	594	[−22; 1187]	4
Metropolitan France	65 834 837	29 400	35 282	42 768	107 450	27 758	[25 905; 29 588]	19	29 541	[27 047; 31 997]	17	25 501	[21 290; 29 645]	10
Réunion Island	873 102	0	35	141	176	−24	[−110; 57]	−2	−126	[−250; −11]	−8	−85	[−288; 103]	−3
Mayotte	310 022	33	15	115	163	108	[−10; 202]	23	19	[−70; 87]	6	210	[57; 327]	42
Martinique	347 686	15	20	54	89	76	[6; 139]	9	190	[108; 265]	20	−23	[−126; 71]	−2
Guadeloupe^b^	418 103	18	155	120	293	53	[−24; 124]	6	262	[168; 348]	27	2	[−112; 104]	0
French Guyana	301 099	61	11	–	72	65	[10; 111]	24	−14	[−73; 33]	−6			
France	68 084 849	29 527	35 518	43 198	108 243	27 638	[25 766; 29 486]	18	29 391	[26 877; 31 870]	17	24 855	[20 598; 29 046]	9

Note: SI-VIC, victim information system in health care institutions; ESMS, social and medico-social institutions; EHPAD, institution for dependent elderly people; INSEE, National Institute of Statistics and Economic Studies.

Sources: Santé publique France/Insee/SI-VIC/COVID-19 EHPAD/ESMS application.

a INSEE estimation, 1 January 2023.

b Guadeloupe included Saint Martin and Saint Barthélemy islands.

### First approach

In total, 29 400 deaths identified as COVID-19 were recorded in HC and MS institutions in metropolitan France during the first epidemic period, 35 282 during the second and 42 768 during the third.

During the first epidemic period, the regions with the highest number of deaths identified as COVID-19 in HC and MS institutions were Île-de-France (*n* = 11 872), Grand-Est (*n* = 5352), Auvergne-Rhône-Alpes (*n* = 2983) and Bourgogne-Franche-Comté (*n* = 1668). During the second epidemic period, the highest numbers of deaths were recorded in Auvergne-Rhône-Alpes (*n* = 8552), Île-de-France (*n* = 4878), Pays-de-la-Loire, Hauts-de-France and Grand-Est (just over 3100 deaths each). During the third epidemic period, it was Île-de-France (*n* = 8274 deaths), Auvergne-Rhône-Alpes (*n* = 5340), followed by Hauts-de-France, Pays-de-la-Loire and Grand-Est (4800–4300 deaths).

If the highest numbers of deaths were observed during the first epidemic period in Île-de-France and Grand-Est and during the second epidemic period in Auvergne-Rhône-Alpes, all other metropolitan regions reached their maximum during the third epidemic. Compared with the first epidemic period, the number of deaths identified as COVID-19 in the third epidemic period was 5 times higher in New Aquitaine, 4.2 times higher in Occitania, 4 times higher in Pays-de-la-Loire, and 3.3 times higher in Normandy.

In the FOD, the surveillance system in MS institutions was deployed but underused. The number of deaths identified as COVID-19 in HC institutions increased with each epidemic period in Réunion Island and Martinique. The highest number of deaths identified as COVID-19 was observed during the first epidemic period in French Guiana (*n* = 61), during the second epidemic period in Guadeloupe (*n* = 155) and during the third epidemic period in Mayotte (*n* = 115).

### Second approach

**Figure 1:**
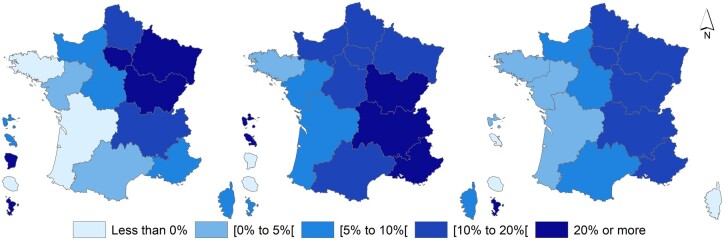
Distribution of excess all-cause mortality for all ages (%) estimated by modelling, for the first three regional epidemic periods of COVID-19, by region, March 2020 to May 2021 (Sources: Santé publique France/Insee)

Overall, more than 27 700 excess deaths from all-causes were estimated in metropolitan France during the first epidemic period, more than 29 500 during the second and 25 500 during the third. There is an east–west gradient in the relative excess of deaths from all-causes (all ages) during each epidemic period, with the Atlantic coastal regions relatively unaffected (figure 1).

The greatest regional disparities were observed during the first epidemic period. The northeast quarter regions were the most affected: Île-de-France with 67% of excess deaths (i.e. +12 443 deaths [12 047; 12 830]), Grand-Est with 38% of excess deaths (i.e. +4880 deaths [4542; 5210]) and Bourgogne-Franche-Comté with 23% of excess deaths (i.e. +1647 deaths [1404; 1882]). The regions of New Aquitaine and Brittany showed a deficit on mortality with −1% (i.e. −229 deaths [−617; 149]) and −3% (i.e. −281 deaths [−543; 26]) respectively.

All metropolitan regions were affected by excesses during the second epidemic period. The largest relative excesses were concentrated in the southeastern quarter (excluding Corsica). Auvergne-Rhône-Alpes recorded +45% excess deaths over the period (i.e. +9011 [8438; 9568] deaths), Bourgogne-Franche-Comté had +30% excess deaths (i.e. +2521 [2196; 2834] deaths), and Provence-Alpes-Côte-d’Azur had 22% (i.e. +3345 [2915; 3762] deaths). Apart from the Île-de-France and Grand-Est regions, which were particularly affected during the first epidemic period, the largest relative excesses in the other metropolitan regions were observed during this second epidemic period.

The relative excesses of all-cause deaths for all ages by region were more homogeneous in the third epidemic period than in the previous ones. Relative excesses ranged from +16% of excess deaths (i.e. +3531 deaths [2809; 4232]) in Provence-Alpes-Côte-d’Azur to +2% of excess deaths (i.e. +252 deaths [−337; 819]) in Brittany. Corsica was the only region with a negative mortality rate, with −1% deaths (i.e. −16 deaths [−216; 160]) during this epidemic period.

Overseas, Guadeloupe and Martinique had a large excess of mortality during their second epidemic period between August and mid-November 2020 (+27%, i.e. +262 deaths [168; 348] and +20%, i.e. +190 deaths [108; 265], respectively). The largest excess mortality was observed during the first epidemic period in French Guiana (+24%, i.e. +65 deaths [10; 111]) and during the third epidemic period in Mayotte (+42%, i.e. +210 deaths [57; 327]). Réunion Island showed an undermortality during each epidemic period.

### Comparison of the two approaches

The excess of all-cause mortality is not explained by the COVID-19 mortality in HC or MS institutions: for each epidemic period and region, the figures observed by the first approach could be higher or lower than those of the second approach. In most metropolitan regions, the number of deaths in HC or MS institutions was higher than the excess of all-cause mortality. Only the Provence-Alpes-Côte-d’Azur region has a number of deaths in HC and MS institutions lower than the excess of all-causes mortality for each epidemic period.

Mortality deficits from all-causes were observed in the first (New Aquitaine, Brittany) and third (Corsica) epidemic periods, while deaths identified with COVID-19 in HC and MS institutions were recorded at the same time. Similarly, Réunion Island did not experience an excess of all-cause mortality during the three epidemic periods, despite the registration of COVID-19-related deaths in the health centres.

In Bourgogne-Franche-Comté, the highest number of COVID-19 identified deaths was observed in the third period, whereas the highest excess of all-cause mortality was recorded in the second epidemic period.

## Discussion

### Completeness and limitations of the data used

#### SI-VIC

HC institutions were legally obliged to complete in this database. The completeness of this source can be considered as excellent. The availability of virological tests evolved during the pandemic, resulting in a lack of virological validation during the first months. In addition, although there was a variable indicating whether the person was hospitalized with COVID-19 or simply a carrier of COVID-19, it showed regional and temporal variations suggesting that its quality was variable in time and space.

#### MS

As the MS system is based on declarations from the institutions, it cannot guarantee either the completeness of its collection or the imputability of the recorded deaths to COVID-19. The completeness of this source may also vary both at regional level and over time. This system has changed over time. A first database was launched on 28 March 2020, with retrospective entry from 1 March 2020.^10^^,^^11^ This was replaced by a second version from 19 March 2021 in order to simplify the declaration and improve the quality of the data and harmonize the indicators produced.^6^ Consistency checks and corrections have been applied to these declarative data.^6^ However, despite these checks, data entry errors may persist. The change of database has also resulted in episodes being closed before the final report is written. In addition, the reporting criteria have changed over time, making it impossible to guarantee consistency of reporting in the system.^6^ Moreover, the MS system is not widely used in the FOD: very few deaths have been counted by this system.

#### INSEE—Deaths from all-causes

As the deaths were collected with a minimum delay of 28 days, they are considered almost exhaustive for the municipalities considered. As most of the computerized municipalities are urban, the comparison between the extrapolation to all the municipalities and the deaths actually observed *a posteriori* may differ slightly.

Two complementary approaches were used to assess regional mortality associated with COVID-19 during the first three epidemic periods.

### First approach

The first approach quantified the number of deaths of patients infected with or diagnosed with COVID-19 that occurred in HC or in MS institutions, ignoring deaths at home. Moreover, the imputability of these deaths to COVID-19 is not established, since people could be tested positive for COVID-19 during their hospital stay, without this infection contributing to their death. Thus, the 107 450 deaths recorded in metropolitan France using this approach reflect an underestimation by place of death and an overestimation by this systematic imputability, the meaning of which is difficult to assess without information on the causes of death.

### Second approach

The second approach made it possible to observe the overall impact, including deaths related to COVID-19, deaths indirectly related, in particular, to difficulties in accessing care, and deaths avoided either by a change in living conditions (lockdown, barrier measures) or because COVID-19 acted as a competing cause of deaths that would have occurred during these periods. This approach therefore only quantifies the impact on general mortality during the different epidemic periods, rather than the share attributable to COVID-19. This approach was similar to work that calculated the impact of heat waves in France, Germany and the USA^12–14^ or, more recently, on COVID-19.^15^ First of all, the expected mortality definition is the crucial point,^16^ regarding especially if we want to avoid all preventable mortality or compare with usual observed ones, i.e. assuming that excess deaths in winter or in summer are inevitable. In this paper, as stated in the methods section, we only refer to the latter, knowing that this will underestimate the overall toll.

The method used was to model mortality to reflect the level usually observed at any time of the year. Other methods exist and have been used, whether for the COVID-19 or for quantifying heat waves. For example, the INSEE^17^ and the National institute for demographic studies^18^ have compared 2020 with 2019, Santé publique France had given first estimates on the months of March to May 2020^19^^,^^20^ based on the Euromomo alarm system.^21^ The comparison with a single year allows to take as a reference the mortality closest in time. However, this estimate is not very robust as mortality varies from year to year, according to, for example, the intensity of epidemics or heat waves, as INSEE points out in the methodological note accompanying its estimate. Thus, Hémon D^22^ used the average of the previous three years to estimate the summer heat wave, in order to avoid year-to-year variability and to avoid comparing years that are too far apart. The choice of the number of years is a compromise between the robustness of a daily average and proximity in order to avoid a long-term trend effect. Euromomo, on the other hand, is an alert system that postulates that the effect of some or some part of the risk factors can be approximated by a regular seasonality defined on spring and autumn, modelled with a sinusoid. Its objective is to warn an unusually high level in the absence of any other risk factors other than this seasonality. It does not reflect the mortality usually observed due to all the factors influencing it (epidemics, meteorology, air pollution, etc), as the methods of comparison with 2019 or the one proposed here do. Its baselines will be lower than corresponding all-cause baselines and the model tends to produce excess mortality on a whole year.^23^ Our approach latter allows to determine the usual mortality over the last five years, whatever the season, by integrating the evolution of the population during this period. This approach also allows, unlike the others, to estimate the uncertainty, related to modelling only, around the excess (or deficit). One main strength of this modelling approach, compared with similar one used by WHO,^24^ is the use of observed data between epidemic waves and after. Our predictions on mortality trend allowing our seasonal but mostly the long-term trend to benefit from these periods to be recalibrated, so avoiding any drift. Our expected mortality can be considered more as interpolated than extrapolated one. So, most of the criticism^25^ linked to the extrapolation of mortality level and trend seem less relevant in our case.

In this system, deaths are reported and recorded by the municipality of death and not by the place of usual residence of the deceased. The periods of lockdown caused population movements.^26^ The deaths of displaced persons may have been registered in a region other than their usual place of residence.

### Complementary approaches

The two approaches were consistent in the most affected regions (according to the epidemic periods). In the regions with lower excess deaths from all-causes, there was greater discrepancy between the number of deaths in HC and MS institutions. An analysis by age group according to the two approaches might allow a better understanding of these differences. Also, we observed that COVID-19 deaths exceeded excess mortality, mainly during the second and third epidemic periods. This trend could be related to several factors. First, testing capacity increases with time, amplifying the systematic attribution of deaths with positive testing, as aforementionned. Second, these epidemics period occurred in winter, period when mortality is usually higher, in connection with influenza, other respiratory viruses or temperature, for example. Expected mortality, what could have been expected if there was no COVID-19, therefore includes the usual effects of these factors. As these respiratory viruses were not circulating,^27^ COVID-19, as a competing cause of death, probably replaced part of their impact.

### Regional disparities

The results revealed regional disparities in mortality associated with COVID-19. The eastern half of metropolitan France was particularly affected by the first two epidemic periods compared with the western half. The proximity to the initial pandemic hotspots in the Alpes could be an explanation of the gradient during the first epidemic period.^28^ The impact of COVID-19 during the different successive epidemic periods was influenced by several factors: the population infected during each period, the circulating strains, vaccination, the application of barrier measures and the sanitary restrictions implemented in each region. For example, vaccination rates in the overseas departments were generally lower than those recorded in the metropolitan region, having a significant impact on hospitalizations^29^ and deaths. The estimation of regional mortality related to COVID-19 will require further work taking into account these different parameters.

The deficit or small excess of deaths from all-causes (all ages) mortality, recorded in the western regions of metropolitan France during the first epidemic period, could be explained by the implementation of strict restrictions (1st lockdown) before the massive spread of the virus in these regions, thus limiting contamination and the associated risk of death. These restrictions probably reduced the spread of other viruses, especially influenza, as observed in Australia, Uruguay and New Zealand.^30^ In addition, strict lockdown has resulted in a drastic reduction of traffic and therefore of air pollution, generating a decrease in deaths related to these two causes.^31^ A significant increase in all-cause, especially non-COVID-19 mortality at home was observed during the first lockdown.^32^ The restriction measures, and in particular the first lockdown (spring 2020), have modified the use of medical care in France. A significant decrease in visits to emergency departments was recorded^33^^,^^34^ suggesting a reduced need to resort to them (fewer travels and associated accidents, application of barrier measures reducing infectious risk^18^ or, on the contrary, the fear of exposure to the virus (e.g. in waiting rooms)).^35^^,^^36^

Depending on the epidemic period, hospitals in some regions have activated their emergency plan (white plan) in order to respond to the need to admit COVID-19 patients to critical care. The associated deprogramming may partly explain the regional disparities and has led to a loss of opportunity for the patients concerned, especially most vulnerable patients (elderly with preexisting comorbidities),^37^ with the potential consequence of an increase in the mortality of these patients, which could also be delayed.^38^

In some regions particularly impacted by COVID-19 cases, the HC system may have led to an increase in mortality from other causes. Without a comparison of specific causes of death, it is difficult to determine whether this effect was actually present in France.

The detailed analysis of medical causes of death, by the CépiDc-Inserm, will make it possible to quantify the excess of deaths attributable to COVID-19 during the various waves of this pandemic. Electronic death registration, which increased significantly during the pandemic, is still insufficient for (near) real-time monitoring of medical causes of death. This complementary data source is still being deployed to physicians and HC and MS institutions. When sufficiently widespread in all regions, it will allow a rapid estimation of mortality related to an emerging pathology such as COVID-19.

## Data Availability

The complete datasets of this research are only available under strictly secured conditions as it contains individual data protected by French legislation. Aggregated data could be shared to the corresponding author, Alain Le Tertre (alain.letertre@santepubliquefrance.fr). Key points This study described de mortality during the first three COVID-19 epidemics. The deaths of patients infected with or diagnosed with COVID-19 recorded in health care or medico-social institutions reflect an underestimation by place of death (leaving out deaths at home) and an overestimation by this systematic imputability, the meaning of which is difficult to assess without information on the causes of death. Several factors (age, difficulties in accessing care, and deaths avoided either by a change in living conditions or because COVID-19 acted as a competing cause of death) influenced the estimates of regional mortality associated with COVID-19.
